# Neutrophil CD64 index for rapid diagnosis of *Pneumocystis jirovecii* pneumonia in malignancy patients requiring mechanical ventilation: a retrospective analysis

**DOI:** 10.3389/fmicb.2026.1706786

**Published:** 2026-01-27

**Authors:** Xiaoming Li, Hongyu Yi, Guixin Wu, Aiting He, Rui Li, Yi Long, Changhai Lin, Zhengying Jiang

**Affiliations:** 1 Department of Critical Care Medicine, Chongqing University Cancer Hospital, Chongqing, China; 2 Department of Critical Care Medicine, General Hospital of Tibet Military Command, Lhasa, China; 3 Department of Laboratory, Chongqing University Cancer Hospital, Chongqing, China

**Keywords:** biomarker, diagnosis, neutrophil CD64, *Pneumocystis jirovecii* pneumonia, prognosis

## Abstract

**Background:**

*Pneumocystis jirovecii* pneumonia (PJP) incidence and associated mortality have risen significantly in non-HIV immunocompromised patients, highlighting the urgent need for rapid, non-invasive diagnostics. Current methods face limitations including invasiveness, prolonged processing, or inadequate specificity. The neutrophil CD64 (nCD64) index emerges as a promising novel biomarker. Here, we conducted this study to evaluate the diagnostic performance of nCD64 index for PJP and further assess the predictive value of its longitudinal changes for 28-day mortality.

**Methods:**

This retrospective cohort study (July 2022–March 2025) analyzed mechanically ventilated malignancy patients with unexplained diffuse pulmonary infiltrates at a tertiary intensive care unit (ICU). PJP diagnosis required predefined clinical, radiological, and bronchoalveolar lavage fluid metagenomic next-generation sequencing (BALF mNGS) criteria. The nCD64 index was measured via flow cytometry at ICU admission and serially after ≥3 days of anti-PJP therapy. Diagnostic performance for PJP and prognostic value for 28-day mortality were assessed.

**Results:**

Among 28 PJP and 38 non-PJP patients, nCD64 index was significantly higher in PJP (13.33 vs. 2.84, *p* < 0.001). Receiver operating characteristic (ROC) curve analysis showed an area under the curve (AUC) of 0.846 (95% CI: 0.736–0.932) for PJP diagnosis, with sensitivity 89.3% and specificity 71.1% at cutoff ≥7. Multivariate analysis confirmed nCD64 index as an independent PJP predictor (OR = 1.097, 95% CI: 1.026–1.173; *p* = 0.007). Post-therapy nCD64 index elevation predicted 28-day mortality with high sensitivity (81.8%) and specificity (86.7%).

**Conclusion:**

The nCD64 index functions as a dual-purpose biomarker for malignancy patients with respiratory failure requiring mechanical ventilation: it provides a rapid, non-invasive diagnostic tool for PJP and dynamically stratifies mortality risk. Moreover, dynamic tracking offers a real-time window into treatment response, guiding therapeutic decisions.

## Introduction

*Pneumocystis jirovecii* pneumonia (PJP) represents a potentially fatal opportunistic infection predominantly affecting immunocompromised hosts. Among patients requiring intensive care unit (ICU) admission for respiratory failure, PJP incidence rises substantially, accounting for approximately 19.2% of suspected or confirmed cases identified during routine diagnostic investigations (Giacobbe et al., 2023). In recent years, the population at risk for PJP has shifted significantly, driven by the widespread use of immunosuppressants, biologic agents, and corticosteroids for treating conditions such as inflammatory diseases and malignancies. Although the incidence of PJP has declined among HIV-infected individuals, it has risen substantially in non-HIV patients, particularly those receiving immunosuppressive therapy (Kanj et al., 2021; Kottom et al., 2023; McMullan et al., 2024). Critically, PJP portends exceedingly high mortality. Research demonstrates that delayed diagnosis and treatment are directly associated with adverse outcomes, emphasizing the paramount importance of timely diagnostic intervention for improving patient survival rates (Roux et al., 2014; Kamel et al., 2024). Therefore, it is important for clinicians to identify potential PJP in time and conduct interventions promptly.

Although Gomori methenamine silver (GMS) staining is a gold standard morphological method for the definitive diagnosis of PJP, specimen collection typically requires invasive procedures (e.g., bronchoalveolar lavage or lung biopsy), increasing patient risk. Furthermore, the staining process is time-consuming, limiting its utility for rapid clinical decision-making. Crucially, sensitivity declines significantly in cases of low pathogen burden, which is characteristically seen in non-HIV immunocompromised patients (Lagrou et al., 2021; Corsi-Vasquez et al., 2019; Szvalb et al., 2020). Polymerase chain reaction (PCR) exhibits high sensitivity and can detect infections with low pathogen burden. However, it cannot distinguish between colonization and infection. This limitation may lead to false-positive diagnoses and unnecessary treatment (Szvalb et al., 2020; Goterris et al., 2019). While metagenomic next-generation sequencing (mNGS) has been increasingly applied in recent years for diagnosing complex infections, including PJP, its high technical complexity, substantial cost, and inability to reliably distinguish between infection, colonization, and contamination limit its clinical utility (Wang et al., 2022; Gu et al., 2019; Theis et al., 2019). Serum (1,3)-*β*-D-glucan (BDG), a non-invasive serological marker, offers readily accessible sample collection and rapid turnaround times. Elevated BDG levels are indicative of fungal cell wall components and demonstrate good sensitivity for PJP screening. However, its lack of specificity for PJP can lead to false-positive results (Giacobbe et al., 2023; Del Corpo et al., 2020; Morjaria et al., 2019).

CD64, a high-affinity IgG Fc receptor, exhibits rapid and strongly inducible expression on neutrophils in response to infection or proinflammatory cytokines IFN-*γ* and granulocyte colony-stimulating factor (G-CSF) (Liu et al., 2022). Neutrophil CD64(nCD64) index demonstrates strong predictive capacity for sepsis, while its longitudinal changes provide significant prognostic utility (Yeh et al., 2019; Pham et al., 2023). We observed significantly elevated nCD64 index in PJP patients during clinical practice. However, dedicated studies evaluating its utility are lacking. Therefore, we conducted this study to evaluate the diagnostic performance of nCD64 index for PJP and further assess the predictive value of its longitudinal changes for 28-day mortality.

## Materials and methods

### Data source and patient selection

This retrospective analysis was performed within the ICU of Chongqing University Cancer Hospital, a tertiary academic medical center located in Chongqing, China. The study examined medical records from critically ill patients admitted to the ICU between July 2022 and March 2025. Ethical approval was granted by the Ethics Committee of Chongqing University Cancer Hospital (CZLL2025-041-001). Informed consent requirements were waived owing to the retrospective study design. The inclusion criteria were as follows: (1) Age ≥ 18 years; (2) Patients with malignancy requiring mechanical ventilation for respiratory failure; (3) Chest computed tomography (CT) demonstrating unexplained diffuse pulmonary infiltrates; (4) Bronchoalveolar fluid (BALF) mNGS performed; (5) BDG and nCD64 index testing performed within 48 h of ICU admission. The exclusion criteria were as follows: (1) Death or discharge within 48 h of ICU admission; (2) Receipt of therapeutic-dose trimethoprim-sulfamethoxazole (TMP-SMX) before BALF mNGS testing; (3) HIV infection; (4) G-CSF administration within 7 days before nCD64 index testing.

Patients were classified into the PJP group if they met all the following criteria: (1) underlying immunosuppression: including use of therapeutic doses of ≥0.3 mg/kg prednisone equivalent for ≥2 weeks in the past 60 days, or a current/expected CD4 + lymphocyte count of <200 cells/μL due to their medical condition or treatment; (2) typical PJP clinical manifestations: including fever and respiratory symptoms such as cough, dyspnea, or hypoxemia; (3) typical PJP-associated radiologic abnormalities on chest CT: showing extensive, bilateral ground-glass opacities, with or without a mosaic pattern, cystic changes, or pneumothorax (Lagrou et al., 2021); and (4) microbiological detection of *P. jirovecii* by BALF mNGS (Lagrou et al., 2021; Wang et al., 2022). Patients who did not meet the above criteria were assigned to the non-PJP group. All patient classifications were independently evaluated by two physicians (XML and GXW). Discrepancies were resolved through adjudication by a third physician (ZYJ).

### Data collection

Clinical data were sourced from electronic medical records, encompassing demographics (age, sex), oncological status (malignancy subtype, comorbidities), Sequential Organ Failure Assessment (SOFA) and Acute Physiology and Chronic Health Evaluation II (APACHE II) scores at ICU admission, admission laboratories, and pre-ICU anti-tumor therapies (chemotherapy/thoracic radiotherapy/immunotherapy within 90 days). Outcomes comprised 28-day mortality, mechanical ventilation duration, ICU stay, and hospital stay. To assess dynamic changes in the nCD64 index and its association with 28-day mortality in PJP patients, we additionally collected the nCD64 index after anti-PJP treatment (for at least 3 days).

### Measurement of the nCD64 index

Peripheral blood samples (2 mL) were collected in EDTA-K₂-anticoagulated vacuum tubes and processed within 24 h. Aliquots of 200 μL whole blood were stained with a pre-mixed antibody cocktail containing the following components: 5 μL CD45-QB500 (Clone HI30), 20 μL CD14-FITC (Clone MEM-15), 20 μL HLA-DR PerCP (Clone HI43), and 20 μL CD64-PE (Clone 10.1; Kuangbo Tongsheng Biotechnology, Tianjin, China). After vortex mixing, samples were incubated for 15 min at room temperature (RT) in the dark. Erythrocytes were lysed by adding 500 μL 1 × FACSLysing Solution (Tongsheng Shidai Biotechnology, Beijing, China) and incubating at 37 °C for 10 min in the dark, followed by two washes with PBS (300 × g, 5 min). Cell pellets were resuspended in 200 μL PBS. Samples were acquired on a BD FACSCanto™ II flow cytometer (BD Biosciences) calibrated daily with standard beads. Data collection utilized a predefined template in BD FACSDiva™ software (v8.1) with a threshold of 800 events set on CD45 to minimize debris interference. Neutrophil gating and median fluorescence intensity (MFI) quantification were performed within the same software.

A sequential gating strategy was employed to identify neutrophil, monocyte, and lymphocyte populations. First, doublets were excluded by gating on single cells using FSC-A vs. FSC-H. Within the singlet gate, leukocytes were identified based on FSC-A and SSC-A characteristics. Neutrophils, monocytes, and lymphocytes were further distinguished using CD14 vs. SSC-A and CD45 vs. SSC-A plots. The MFI of CD64 expression on neutrophils, lymphocytes, and monocytes in the stained samples were measured. The nCD64 index was calculated using the following formula: nCD64 index = [MFI CD64 (neutrophils)/MFI CD64 (lymphocytes)]/[MFI CD64 (monocytes)/MFI CD64 (neutrophils)]. The established normal reference range for this index is 0–1.28. Representative flow cytometry plots illustrating the gating strategy and CD64 expression histograms are provided in Supplementary material.

### Statistical analysis

Continuous variables are presented as median and interquartile range (IQR), with intergroup differences analyzed using the Mann–Whitney U test (effect sizes reported as Hodges-Lehmann median differences and 95% CIs); categorical variables are expressed as frequencies and percentages, compared by Pearson’s chi-square or Fisher’s exact test (>20% expected cell frequencies <5). The predictive capacity of nCD64 index for PJP was evaluated through receiver operating characteristic (ROC) curve analysis, calculating the area under the curve (AUC) with DeLong’s 95% CI. Optimal cutoff values were determined by maximizing the Youden index. Sensitivity, specificity, positive predictive value (PPV), negative predictive value (NPV) and accuracy were derived from contingency tables. To find whether nCD64 index was the independent risk factor for PJP, univariate logistic regression analyses and multivariate logistic regression analyses using the “Forward Conditional” method were performed for the variables that showed statistically significant differences between the PJP group and the non-PJP group. Analyses used SPSS 26.0 (Chicago, USA) and MedCalc 20.1 (Ostend, Belgium), with two-tailed *p* < 0.05 defining significance.

## Results

### Patient selection and baseline characteristics

**Figure 1:**
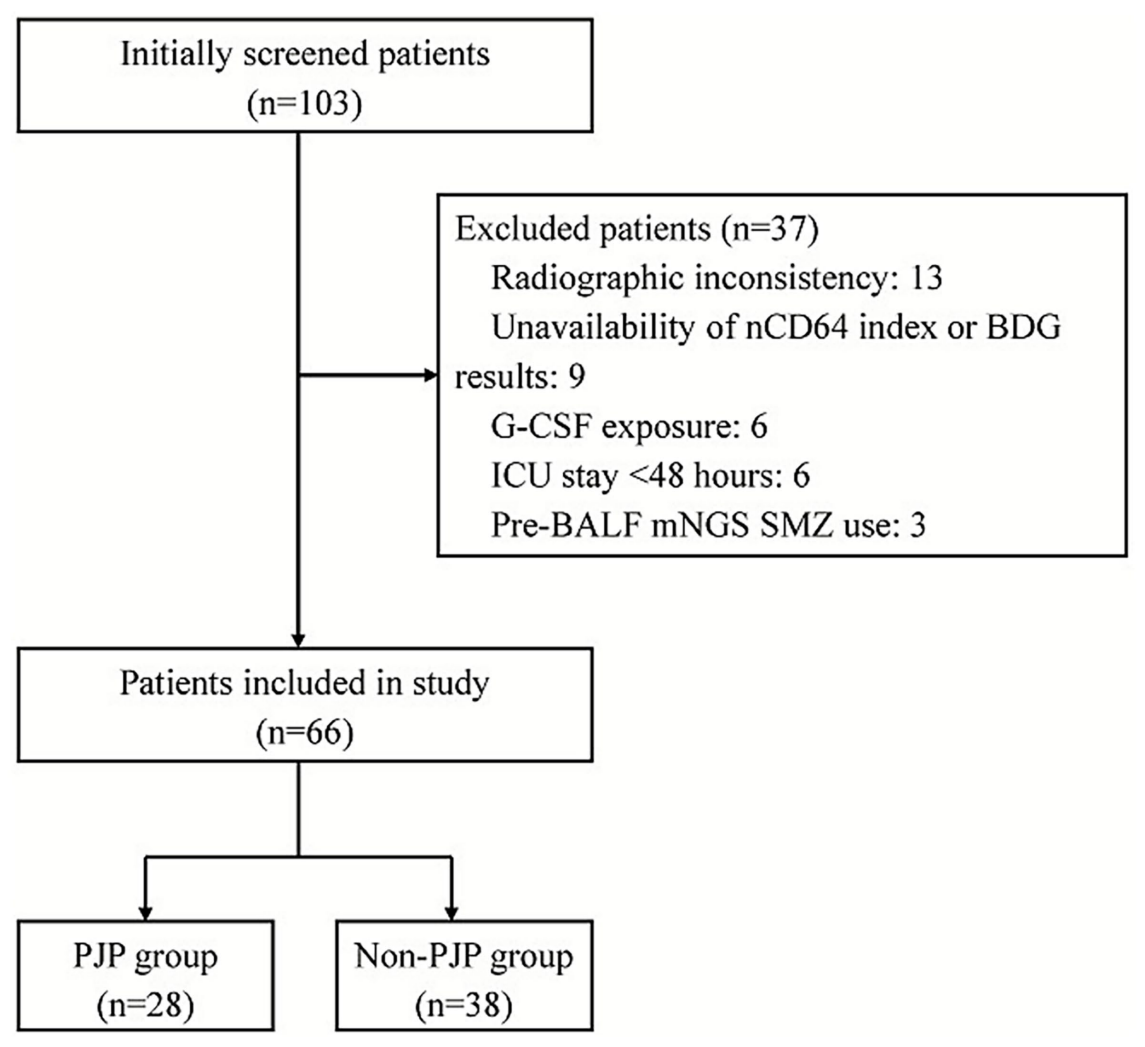
Flowchart of the enrolled patients.

According to our inclusion and exclusion criteria, a total of 66 patients were enrolled during the study period, comprising 28 confirmed PJP cases and 38 non-PJP controls (Figure 1). All patients diagnosed with PJP received anti-PJP therapy during their ICU stay, with TMP-SMX as the first-line treatment regimen. Treatment adjustments, including the use of second-line agents, were made according to clinical response, drug intolerance, or physician discretion. Baseline characteristics are summarized in Table 1. The cohorts were well-matched for age (67.5 vs. 67.5 years, *p* = 0.654), sex (85.7% vs. 84.2% male, *p* = 1.000), and disease severity scores (SOFA 5 vs. 4, *p* = 0.357; APACHE II 22 vs. 21, *p* = 0.221). PJP patients exhibited higher rates of chronic pulmonary disease (39.3% vs. 13.2%, *p* = 0.014) and recent thoracic radiotherapy (50.0% vs. 10.5%, *p* < 0.001), alongside elevated nCD64 index (13.33 vs. 2.84, *p* < 0.001), BDG positivity (50.0% vs. 10.5%, *p* < 0.001), and lactate dehydrogenase (LDH) levels (530.00 vs. 391.50 U/L, *p* < 0.001). Notably, PJP patients demonstrated greater immunosuppression, reflected by lower CD4^+^ T-cell counts (85.50 vs. 205.00 cells/μL, *p* = 0.001) and higher recent chemotherapy exposure (75.0% vs. 50.0%, *p* = 0.040). No intergroup differences existed in white blood cell (WBC), platelet (PLT), interleukin-6(IL-6), procalcitonin (PCT), neutrophil-to-lymphocyte ratio (NLR), C-reactive protein (CRP), or PaO₂/FiO₂ ratio (*p* > 0.05). Despite similar 28-day mortality (46.4% vs. 50.0%, *p* = 0.774), PJP cases had shorter ICU stays (180.00 vs. 238.00 h, *p* = 0.039).

**Table 1:** Baseline characteristics of the study population.

Characteristics [median (IQR) or *n* (%)]	PJP patients (*n =* 28)	Non-PJP patients (*n =* 38)	*p*-value
Demographics			
Age, years (IQR)	67.5[57.25, 70.00]	67.5[58.00,73.00]	0.654
Male (%)	24 (85.7)	32 (84.2)	1.000
SOFA (IQR)	5 [4, 7]	4 [4, 8]	0.357
APACHE II	22 [20, 25]	21 [18, 23]	0.221
Medical history (%)			
Hematological malignancy	4 (14.3)	4 (10.5)	0.935
Solid tumor	24 (85.7)	34 (89.5)	
Comorbidities (%)			
Hypertension	7 (25.0)	7 (18.4)	0.518
Diabetes	7 (25.0)	6 (15.8)	0.352
CHD	7 (25.0)	5 (13.2)	0.218
Chronic pulmonary disease	11 (39.3)	5 (13.2)	0.014
Liver disease	2 (7.1)	3 (7.9)	1.000
Renal disease	1 (3.6)	1 (2.6)	1.000
First laboratory tests			
BDG positive (%)	14 (50.0)	4 (10.5)	<0.001
nCD64 index	13.33 [8.93, 38.95]	2.84 [1.68, 10.85]	<0.001
WBC (*10^9^/L)	8.27 [6.47, 13.52]	12.03 [8.23, 15.24]	0.097
IL-6 (pg/ml) (missing = 3)	294.6 [98.05, 507.10]	150.60 [74.99, 757.30]	0.407
PCT (ng/ml)	0.46 [0.23, 3.17]	0.41 [0.15, 1.67]	0.448
NLR	18.54 [7.08, 34.55]	18.60 [10.19, 35.94]	0.707
CRP (mg/dl) (missing = 10)	158.93 [87.70, 199.99]	122.47 [52.55, 183.49]	0.204
Hb (g/L)	97.00 [82.00, 111.00]	102.50 [79.75, 113.50]	0.825
PLT (*10^9^/L)	153.50 [90.00, 221.00]	191.50 [114.00, 265.50]	0.084
LDH(U/L)	530.00 [490.55, 698.38]	391.50 [246.68, 476.33]	<0.001
PaO2/FiO2(mmHg)	99.15 [64.35, 130.75]	82.15 [55.18, 181.25]	0.851
CD4^+^ T cell (/μL)	85.50 [49.50, 149.25]	205.00 [123.00, 415.25]	0.001
Previous chemotherapy (%) (within 90 days)	21 (75.0)	19 (50.0)	0.040
Previous thoracic radiotherapy (%) (within 90 days)	14 (50.0)	4 (10.5)	<0.001
Previous immunotherapy (%) (within 90 days)	17 (60.7)	14 (36.8)	0.055
28-day mortality (%)	13 (46.4)	19 (50.0)	0.774
Length of stay			
ICU length of stay, hours	180.00 [110.25, 253.75]	238.00 [167.50, 343.25]	0.039
Hospital length of stay, hours	477.00 [391.00,661.75]	560.50 [380.25, 716.25]	0.712
Duration of mechanical ventilation, hours	124.50 [83.25, 202.75]	154.50 [104.75, 273.50]	0.086

IQR, interquartile range; PJP, *Pneumocystis jirovecii* pneumonia; SOFA Sequential Organ Failure Assessment; APACHEII, Acute Physiology And Chronic Health Evaluation II; CHD, coronary heart disease; BDG, (1,3)-β-d-glucan; nCD64, neutrophil CD64; WBC, white blood cell; IL-6, interleukin-6; PCT, procalcitonin; NLR, neutrophil-to-lymphocyte ratio; CRP, C-reactive protein; Hb, hemoglobin; PLT, platelet; LDH, lactate dehydrogenase; ICU, intensive care unit.

### Independent risk factors for PJP identified by regression analysis

Univariate analysis identified six significant predictors of PJP (*p* < 0.05): elevated nCD64 index (OR = 1.111, 95%CI: 1.014–1.179; *p* = 0.001), BDG positivity (OR = 8.500, 95%CI: 2.378–30.377; *p* = 0.001), increased LDH (OR = 1.005, 95%CI: 1.002–1.008; *p* = 0.003), reduced CD4^+^ T-cell counts (OR = 0.996, 95%CI: 0.992–0.999; *p* = 0.016), chronic pulmonary disease (OR = 5.500, 95%CI: 1.523–19.860; *p* = 0.009), and recent thoracic radiotherapy (OR = 8.500, 95%CI: 2.378–30.377; *p* = 0.001).

Multivariate analysis confirmed that nCD64 index remained an independent predictor after adjusting for confounders (OR = 1.097, 95%CI: 1.026–1.173; *p* = 0.007). Thoracic radiotherapy also retained significance (OR = 7.662, 95%CI: 1.769–33.195; *p* = 0.006), while BDG positivity showed borderline association (OR = 4.868, 95%CI: 0.976–24.276; *p* = 0.054). Other variables (LDH, CD4^+^ T cell, chronic pulmonary disease, chemotherapy) did not enter the final model (Table 2).

**Table 2:** Univariate and multivariate logistic regression analyses of risk factors associated with infection of PJP.

Variables	Univariate analysis		Multivariate analysis	
	Unadjusted OR (95%CI)	*P*-value	Adjusted OR (95%CI)	*P*-value
nCD64 index	1.111 (1.014,1.179)	0.001	1.097 (1.026,1.173)	0.007
BDG positive	8.500 (2.378, 30.377)	0.001	4.868 (0.976, 24.276)	0.054
LDH(U/L)	1.005 (1.002,1.008)	0.003		
CD4^+^ T cell(/μL)	0.996 (0.992,0.999)	0.016		
Chronic pulmonary disease	5.5000 (1.523, 19.860)	0.009		
Previous chemotherapy (within 90 days)	3.000 (1.033,8.710)	0.043		
Previous thoracic radiotherapy (within 90 days)	8.500 (2.378, 30.377)	0.001	7.662 (1.769, 33.195)	0.006

PJP, *Pneumocystis jirovecii* pneumonia; nCD64, neutrophil CD64; BDG, (1,3)-β-d-glucan; OR, odds ratio; CI, confidence interval; LDH, lactate dehydrogenase.

### Diagnostic performance of nCD64 index for PJP

**Figure 2:**
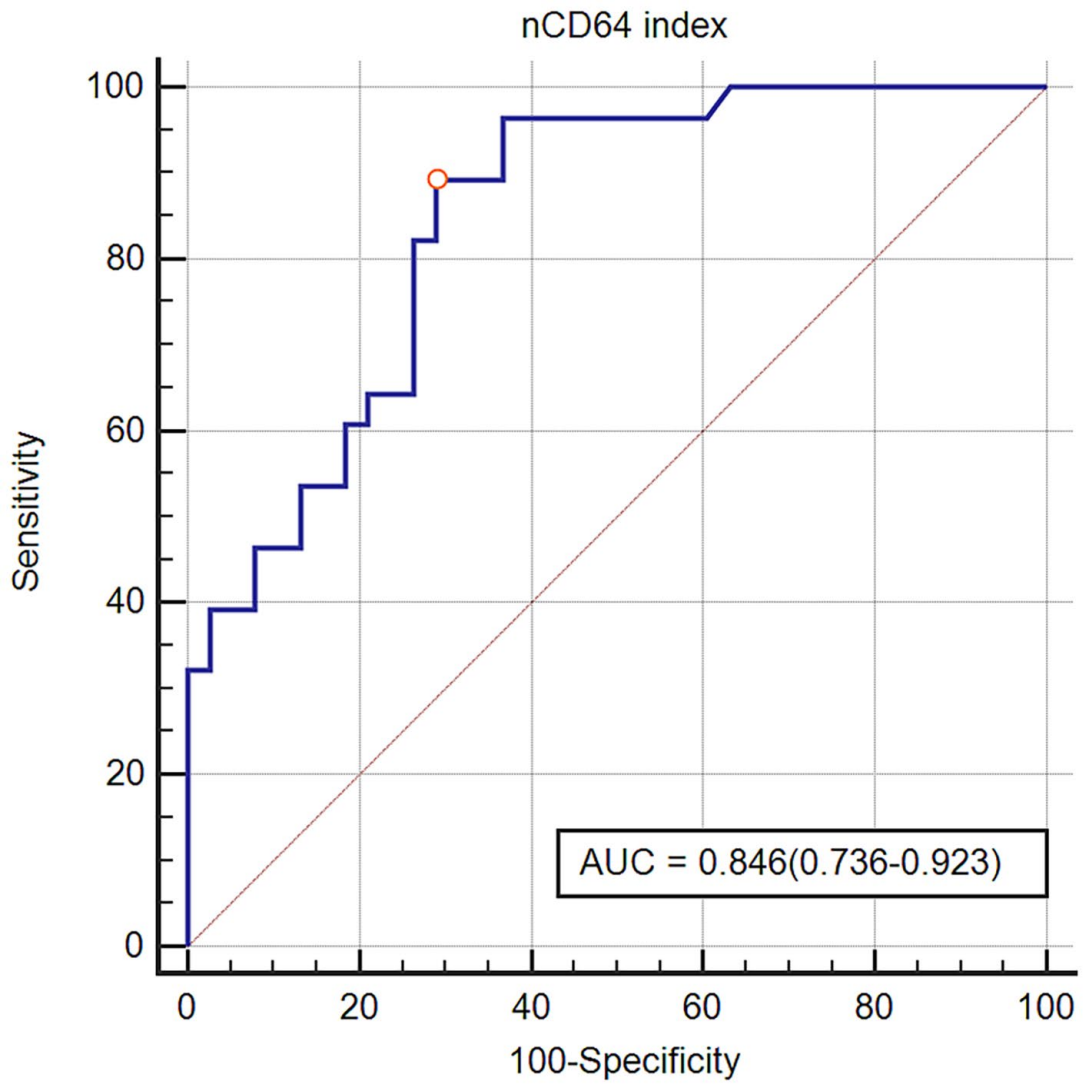
Diagnostic performance of nCD64 index for *Pneumocystis jirovecii* pneumonia in the study population. Receiver operating characteristic curve analysis demonstrates discriminative capacity of nCD64 index, yielding an area under the curve of 0.846 (95% CI: 0.736–0.923).

The diagnostic efficacy of the nCD64 index for PJP was evaluated using ROC curve analysis. As illustrated in Figure 2, the nCD64 index demonstrated a significant discriminatory capacity with an AUC of 0.846 (95% CI: 0.736–0.923). Based on the maximum Youden index, the optimal cut-off value for the nCD64 index was 6.77. For clinical applicability, a rounded cut-off ≥7 was adopted, which yielded identical sensitivity and specificity in our cohort. At this threshold (Table 3), the nCD64 index exhibited a sensitivity of 89.3% and a specificity of 71.1%. The PPV and NPV were 69.4 and 90.0%, respectively, with an overall diagnostic accuracy of 78.9%. Comparative analysis with BDG testing revealed distinct performance characteristics. BDG positivity alone showed lower sensitivity (50.0%) but higher specificity (89.5%) and PPV (77.8%) than nCD64 index. The combination of nCD64 index ≥7 and BDG positivity further improved specificity to 97.4% and PPV to 92.9%, albeit with reduced sensitivity (46.4%).

**Table 3:** Performance of BDG and nCD64 index in predicting infection of PJP.

Cut-off	TP	FP	TN	FN	Sensitivity (%)	Specificity (%)	PPV (%)	NPV (%)	Accuracy (%)
CD64 ≥ 7	25	11	27	3	89.3	71.1	69.4	90.0	78.9
BDG positive	14	4	34	14	50.0	89.5	77.8	70.8	72.7
CD64 ≥ 7 and BDG positive	13	1	37	15	46.4	97.4	92.9	71.2	75.8

PJP, *Pneumocystis jirovecii* pneumonia; BDG, (1,3)-β-d-glucan; nCD64, neutrophil CD64; TP, true positive; FP, false positive; TN, true negative; FN, false negative; PPV, positive predictive value; NPV, negative predictive value.

### Prognostic value of serial nCD64 index changes for 28-day mortality in PJP patients

Serial monitoring of nCD64 index was performed in 26 PJP patients who received at least 72 h of anti-PJP therapy, among whom 11 patients died within 28 days, corresponding to a mortality rate of 42.3%. Post-therapy nCD64 index elevation was observed in 11 patients (42.3%), 9 of whom (81.8%) succumbed to 28-day mortality. Conversely, among the 15 patients (57.7%) with decreased nCD64 index, only 2 (13.3%) experienced fatal outcomes. As summarized in Table 4, the elevation of nCD64 index following anti-PJP therapy demonstrated significant prognostic utility for 28-day mortality. This parameter achieved a sensitivity of 81.8% (9/11) and specificity of 86.7% (13/15), with PPV and NPV of 81.8% (9/11) and 86.7% (13/15), respectively. The overall prognostic accuracy was 84.6% (22/26), indicating that serial nCD64 index assessment provides clinically relevant stratification of mortality risk in PJP patients undergoing treatment.

**Table 4:** Prognostic value of serial nCD64 index changes for 28-day mortality in PJP patients.

TP	FP	TN	FN	Sensitivity (%)	Specificity (%)	PPV (%)	NPV (%)	Accuracy (%)
9	2	13	2	81.8	86.7	81.8	86.7	84.6

PJP, *Pneumocystis jirovecii* pneumonia; nCD64, neutrophil CD64; TP, true positive; FP, false positive; TN, true negative; FN, false negative; PPV, positive predictive value; NPV, negative predictive value.

## Discussion

The principal findings of this study demonstrate the dual clinical utility of nCD64 index in critically ill malignancy patients with suspected PJP. First, the nCD64 index exhibited robust diagnostic performance for PJP, with significantly elevated levels in confirmed cases compared to non-PJP controls. ROC analysis revealed an AUC of 0.846 (95% CI: 0.736–0.923), and at an optimal cutoff of ≥7, it achieved a sensitivity of 89.3% and specificity of 71.1%. Second, longitudinal assessment of nCD64 index dynamics provided potent prognostic stratification for 28-day mortality in PJP patients. Those exhibiting post-treatment nCD64 index elevation had significantly higher mortality, with 81.8% sensitivity and 86.7% specificity for fatal outcome prediction. These results collectively establish the nCD64 index as both a rapid diagnostic biomarker and a dynamic prognostic tool in this high-risk population.

Malignancy patients are highly susceptible to PJP due to profound immunosuppression resulting from both the underlying disease and anti-tumor therapy. This immunosuppression impairs host defense mechanisms, including lymphocyte function, which is a key risk factor for PJP development. Specifically, immunosuppression reduces the ability to mount effective immune responses, facilitating PJP colonization and subsequent pneumonia (Azoulay et al., 2018; Jiang et al., 2025). Malignancy constitutes the predominant predisposing factor among patients hospitalized with PJP, comprising 46.0–55.7% of cases. PJP can trigger excessive lung inflammation and acute respiratory distress syndrome (ARDS), leading to respiratory failure requiring mechanical support, which is associated with a high mortality rate (Gaborit et al., 2019; Sayson et al., 2024). Moreover, delay in antibiotic treatment was associated with accelerated mortality (Kamel et al., 2024; Azoulay et al., 2018). Therefore, for patients with malignant tumors accompanied by respiratory failure who require mechanical ventilation, a group with a high incidence and mortality rate of PJP, early diagnosis and early treatment are of crucial importance.

CD64 is minimally expressed on quiescent neutrophils but undergoes rapid upregulation mediated by proinflammatory cytokines (e.g., IFN-*γ*, G-CSF, TNF-*α*) during microbial challenge and plays an important role in the immune response to infection (Wang et al., 2015; Dimoula et al., 2014). In PJP, *P.jirovecii* induces an alveolar macrophage-dominated inflammatory cascade characterized by chemokine-mediated neutrophil recruitment and sustained proinflammatory cytokine release (Sayson et al., 2024; Liu et al., 2020). These cytokines potently stimulate CD64 expression on infiltrating neutrophils. Malignancy-associated immune compromise further dysregulates this response, impairing pathogen clearance while exacerbating inflammation. PJP frequently progresses to ARDS, particularly in critically ill patients requiring mechanical ventilation, with reported incidences as high as 88.2% in this population (Giacobbe et al., 2023; Kamel et al., 2024). And prior research in patients with acute pancreatitis has demonstrated a strong association between a high nCD64 index and the development of ARDS (Shao et al., 2025), supporting the biological plausibility of our observation in PJP. Thus, the elevated nCD64 index in mechanically ventilated malignancy patients with PJP reflects the convergence of: *P. jirovecii*-specific neutrophil priming, respiratory failure-driven inflammatory cascades, and malignancy-associated immune remodeling. Our findings demonstrate that the nCD64 index serves as a highly sensitive biomarker (89.3%) for rapid PJP diagnosis in malignancy patients with respiratory failure requiring mechanical ventilation, albeit with moderate specificity (71.1%). This performance notably surpasses traditional biomarkers such as BDG, which exhibited significantly lower sensitivity (50.0%) despite higher specificity (89.5%) in our cohort. The low sensitivity of BDG aligns with prior findings in non-HIV immunocompromised populations, showing sensitivities of 35.6 to 53.9% at varying cut-offs. This is attributed to low fungal loads, resulting in a high false-negative rate (Szvalb et al., 2020). Compared to mNGS, nCD64 index offers distinct advantages in speed and accessibility. While mNGS achieves exceptional sensitivity and specificity for *P. jirovecii* detection (Wang et al., 2022; Jiang et al., 2021; Liu et al., 2023), it requires 24–48 h for processing and is costly. Critically, mNGS cannot differentiate colonization from active infection, potentially leading to overdiagnosis. In contrast, nCD64 index results are obtainable within 4 h via flow cytometry, and its elevation reflects active host inflammatory responses rather than mere pathogen presence. The combination strategy (nCD64 index ≥7 + BDG positivity) achieved remarkable specificity (97.4%) and PPV (92.9%), outperforming either test alone. This synergy addresses key limitations of single marker approaches and aligns with recommendations for multi-biomarker algorithms in critically ill patients (Giacobbe et al., 2023). While nCD64 index provides a non-invasive, rapid alternative results, its non-specificity in bacterial co-infections or sterile inflammation necessitates integration with clinical context (Liu et al., 2022).

We also acknowledge that bacterial infections can upregulate nCD64 expression, potentially confounding the interpretation of nCD64 index elevation. However, in our cohort, key systemic inflammatory markers of bacterial infection—including WBC, PCT, IL-6, and CRP—did not differ significantly between PJP and non-PJP groups. This suggests that the burden of bacterial co-infection was comparable between groups and unlikely to be the primary driver of the observed nCD64 index differences. The persistence of nCD64 index as an independent PJP predictor after multivariate adjustment further supports its utility in discriminating PJP from non-PJP causes of respiratory failure in this high-risk population. Nevertheless, in clinical practice, distinguishing PJP from bacterial pneumonia or mixed infections remains challenging. Clinicians should interpret nCD64 index values in conjunction with clinical context and additional biomarkers to differentiate PJP from bacterial pneumonias. And future studies incorporating comprehensive microbiological workup and longitudinal biomarker profiling will be valuable to further clarify the specificity of nCD64 index in PJP diagnosis.

To our knowledge, early detection and effective intervention are critical for reducing PJP-associated mortality. However, emerging resistance to frontline regimens is compounded by the absence of standardized antimicrobial susceptibility testing, as *P. jirovecii* remains unculturable *in vitro*. Consequently, therapeutic monitoring relies solely on clinical indicators—including radiographic resolution and nonspecific biomarkers—with no objective measures available to quantify treatment response (Held and Wagner, 2011). During initial therapy, conventional biomarker BDG may exhibit transient elevation attributable to *P.jirovecii* lysis. Consequently, serial BDG levels cannot reliably reflect therapeutic response (Watanabe et al., 2009). Icardi et al. reported that the nCD64 index responded to antibiotic therapy effectiveness and correlated with the improvement of sepsis symptoms, as patients receiving adequate antibiotic therapy showed a quick reduction in their nCD64 index after 2 to 3 days (Icardi et al., 2009). The studies conducted by Dimoula et al. and Ghosh et al. revealed similar results (Dimoula et al., 2014; Ghosh et al., 2018). This distinct dynamic pattern suggests that serial nCD64 index monitoring may serve as a biomarker for both therapeutic response and clinical recovery during ICU management. Our research demonstrates that the dynamic trajectory of the nCD64 index after initiating anti-PJP treatment can effectively predict the 28-day mortality. Mechanistically, the increase in nCD64 index levels may indicate a lack of response to the first-line treatment and necessitates the timely switching to a second-line treatment regimen. At minimum, such increases warrant vigilant monitoring including chest CT re-evaluation for radiographic progression. Defining the prognostic value of serial nCD64 dynamics may enable personalized clinical management—facilitating early treatment escalation in non-responders—to improve outcomes for critically ill patients with PJP. However, these findings need to be validated in larger-scale, multi-center cohorts.

It is noteworthy that our study identified recent thoracic radiotherapy as an independent risk factor for PJP, with the following potential underlying mechanisms: Systemic immunosuppression driven by recruitment of immunosuppressive regulatory cells, inhibition of cytokine networks, structural damage to primary (bone marrow, thymus) and secondary (spleen) lymphoid organs, and direct depletion of circulating lymphocytes—exacerbated by substantial cardiopulmonary blood pool irradiation (Abravan et al., 2020); Local pulmonary compromise via radiation pneumonitis-induced alveolar epithelial damage, inflammatory infiltration, and impaired mucociliary clearance, creating a permissive microenvironment for *P. jirovecii* colonization (Mei et al., 2022; Chen et al., 2023); and secondary immunosuppression arising from corticosteroid-dependent management of radiation pneumonitis (particularly moderate–severe cases), which independently elevates opportunistic infection risk (Voruganti Maddali et al., 2024). Collectively, radiotherapy-induced impairment of pulmonary defenses and compounded immunosuppression critically undermine pathogen clearance, ultimately predisposing to PJP.

Moreover, chronic pulmonary disease—primarily chronic obstructive pulmonary disease (COPD)—was more prevalent in PJP patients. COPD may predispose individuals to PJP through several mechanisms: impaired mucociliary clearance and alveolar macrophage dysfunction facilitate *P. jirovecii* colonization; chronic airway inflammation and epithelial injury create a permissive microenvironment for opportunistic pathogens; and frequent corticosteroid use for COPD exacerbations further suppresses cellular immunity. These factors collectively increase the risk of PJP. This is the first study to establish the nCD64 index as a dual-role biomarker—providing both diagnostic and prognostic utility—in mechanically ventilated malignancy patients with PJP, a high-mortality cohort. Moreover, dynamic monitoring of the nCD64 index offers a real-time window into treatment response, guiding subsequent therapeutic decisions. However, this single-center retrospective design and moderate sample size limit generalizability and preclude subgroup analyses. External validation in diverse cohorts is required before clinical implementation.

## Conclusion

This is the first study to establish the nCD64 index as a dual-role biomarker—providing both diagnostic and prognostic utility—in mechanically ventilated malignancy patients with PJP. For diagnosis, it demonstrated robust performance and independent predictive value. For prognosis, serial monitoring revealed that post-treatment nCD64 elevation strongly predicted 28-day mortality. Moreover, dynamic tracking offers a real-time window into treatment response, guiding therapeutic decisions. However, further validation in larger multicenter cohorts is warranted.

## Data Availability

The raw data supporting the conclusions of this article will be made available by the authors, without undue reservation.

## Glossary

AUC: area under the curve
ARDS: acute respiratory distress syndrome
APACHE II: Acute Physiology and Chronic Health Evaluation II
BDG: (1,3)-*β*-D-glucan
BALF: bronchoalveolar lavage fluid
CT: computed tomography
CRP: C-reactive protein
CIs: confidence intervals
COPD: chronic obstructive pulmonary disease
G-CSF: granulocyte colony-stimulating factor
GMS: Gomori methenamine silver
IQR: interquartile range
ICU: intensive care unit
IL-6: interleukin-6
LDH: lactate dehydrogenase
mNGS: metagenomic next-generation sequencing
MFI: median fluorescence intensity
nCD64: neutrophil CD64
NPV: negative predictive value
NLR: neutrophil-to-lymphocyte ratio
PJP: *Pneumocystis jirovecii* pneumonia
PCR: polymerase chain reaction
PPV: positive predictive value
PCT: procalcitonin
ROC: receiver operating characteristic
SOFA: Sequential Organ Failure Assessment
SIRS: systemic inflammatory response syndrome
TMP-SMX: trimethoprim-sulfamethoxazole
WBC: white blood cell
