# Supplementary material for: Neutrophil CD64 index for rapid diagnosis of Pneumocystis jirovecii pneumonia in malignancy patients requiring mechanical ventilation: a retrospective analysis
Source: Front Microbiol. 2026 Jan 27;17:1706786. doi: 10.3389/fmicb.2026.1706786 (PMC12886426; doi:10.3389/fmicb.2026.1706786)
Supplement: Supplementary file 1 [file Table_1.DOC]

**Representative flow cytometry plots and gating strategy for**

**nCD64 index measurement**

A. Under "All Events", plot FSC-A against FSC-H to set gate P1 (excluding doublets), as shown in the figure below.


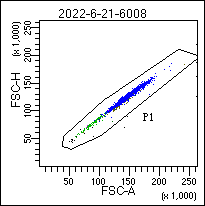


B. Within gate P1, a scatter plot of SSC-A versus FSC-A is created to define the leukocyte gate (WBC gate), as shown in the figure below.


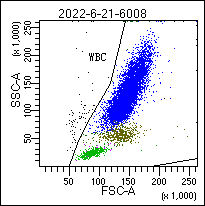


C. Within the WBC gate, a scatter plot of CD14 versus SSC is used to delineate the neutrophil (Neu), monocyte (Mon), and lymphocyte/basophil (P2) populations, respectively, as shown in the figure below.


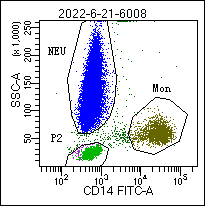


D. Within the P2 gate, a plot of SSC versus CD45 is created to define the lymphocyte gate, as shown in the figure below.


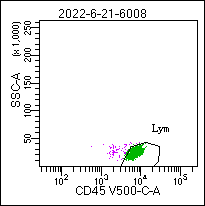


E. Histograms are generated by plotting CD64 versus Count to depict the expression profiles for monocytes, neutrophils, and lymphocytes, respectively.


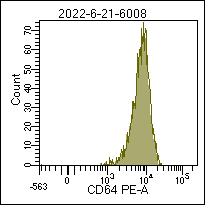

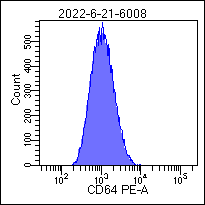

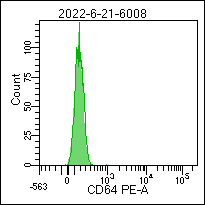


monocytes neutrophils lymphocytes

F. Using neutrophil HLA-DR expression as a negative control, a quadrant gate is applied within the monocyte population to determine the HLA-DR positivity rate of monocytes


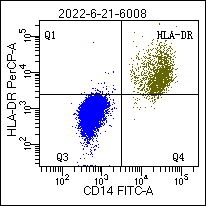


G. By selecting the three graphs from step E and exporting the data, the proportions and mean fluorescence intensity (MFI) values for monocytes (Mon), neutrophils (Neu), and lymphocytes (Lym), as well as the HLA-DR positivity rate of monocytes (Q2), are obtained.


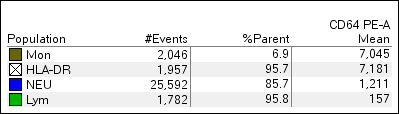


H. nCD64 index = [MFI CD64 (neutrophils) / MFI CD64 (lymphocytes)] / [MFI CD64 (monocytes) / MFI CD64 (neutrophils)]
